# Neural expression and post-transcriptional dosage compensation of the steroid metabolic enzyme 17β-HSD type 4

**DOI:** 10.1186/1471-2202-11-47

**Published:** 2010-04-01

**Authors:** Sarah E London, Yuichiro Itoh, Valentin A Lance, Petra M Wise, Preethika S Ekanayake, Randi K Oyama, Arthur P Arnold, Barney A Schlinger

**Affiliations:** 1 Interdepartmental Program in Neuroscience, University of California at Los Angeles, Los Angeles, CA, USA; 2 Department of Physiological Science, University of California at Los Angeles, Los Angeles, CA, USA; 3 Laboratory of Neuroendocrinology of the Brain Research Institute, University of California at Los Angeles, Los Angeles, CA, USA; 4 Department of Ecology and Evolutionary Biology, University of California at Los Angeles, Los Angeles, CA, USA; 5 Institute for Genomic Biology, University of Illinois at Urbana-Champaign, Urbana, IL, USA

## Abstract

**Background:**

Steroids affect many tissues, including the brain. In the zebra finch, the estrogenic steroid estradiol (E_2_) is especially effective at promoting growth of the neural circuit specialized for song. In this species, only the males sing and they have a much larger and more interconnected song circuit than females. Thus, it was surprising that the gene for 17β-hydroxysteroid dehydrogenase type 4 (HSD17B4), an enzyme that converts E_2 _to a less potent estrogen, had been mapped to the Z sex chromosome. As a consequence, it was likely that HSD17B4 was differentially expressed in males (ZZ) and females (ZW) because dosage compensation of Z chromosome genes is incomplete in birds. If a higher abundance of HSD17B4 mRNA in males than females was translated into functional enzyme in the brain, then contrary to expectation, males could produce less E_2 _in their brains than females.

**Results:**

Here, we used molecular and biochemical techniques to confirm the HSD17B4 Z chromosome location in the zebra finch and to determine that HSD17B4 mRNA and activity were detectable in the early developing and adult brain. As expected, HSD17B4 mRNA expression levels were higher in males compared to females. This provides further evidence of the incomplete Z chromosome inactivation mechanisms in birds. We detected HSD17B4 mRNA in regions that suggested a role for this enzyme in the early organization and adult function of song nuclei. We did not, however, detect significant sex differences in HSD17B4 activity levels in the adult brain.

**Conclusions:**

Our results demonstrate that the HSD17B4 gene is expressed and active in the zebra finch brain as an E_2 _metabolizing enzyme, but that dosage compensation of this Z-linked gene may occur via post-transcriptional mechanisms.

## Background

Steroids have profound effects on the brain. In the zebra finch (*Taeniopygia guttata)*, regulation of the estrogenic steroid estradiol (E_2_) within the zebra finch brain may have functional consequences for song behavior. In zebra finches, males sing but females do not. Males also have a larger and more interconnected set of brain areas, or nuclei, specialized to learn and produce song than females [1]. Early in development, E_2 _masculinizes the song circuit required for song production and in adulthood, the function of song areas can be altered by changes in E_2 _after song experience [2-5].

The gonads do not supply the steroids that direct masculinization of the song circuit, but the brain may be an essential source of E_2 _both during development and in adulthood [6-24]. It is still unclear, however, how steroids produced in the brain act on the song system [17,24-28]. Therefore, two non-exclusive theories have been proposed to explain the origin of sex differences in this system: sex differences are caused by local differences in steroid synthesis in or near the song control circuit [6,7,11-13,17], and sex differences are caused by the differential effect of genes localized to sex chromosomes [29,30].

Evidence exists to support both theories. Many of the major steroid -producing and -transducing factors that can act on the song circuit are present in the zebra finch brain [6,7,10,11,18,19,31-37]. For example, early developmental E_2 _administration to females acts in the brain to induce a masculine song circuit and the closer the source of steroids is to a song nucleus the more potent its effect, cultured brain slices produce E_2 _and manipulations of E_2 _levels and estrogen receptor action in these slices prevent full masculinization of song circuitry, and the E_2_-synthetic enzyme aromatase is present in presynaptic terminals within major song nuclei; in fact, in adult male zebra finches, the brain is the major source of E_2 _[2,8,9,15,16,38-42]. On the other hand, *in vivo *manipulations of E_2 _synthesis and action in males does not consistently significantly de-masculinize the song system, the song circuit in genetic females is not masculinized by the presence of functional steroid-secreting testes, and the song nuclei within the male hemisphere of a naturally-occurring gynandromorph (one half genetically male, one half genetically female) are larger than those in the genetically female hemisphere [22,25,28,42-46]. Although in some cases, these findings are partially consistent with the neural steroid synthesis hypothesis, they primarily suggest that genetic differences between males and females contribute to the masculine development of the song system.

Indeed, in mammals and in zebra finches, data is accumulating that male brain cells are fundamentally different than female cells by virtue of their different complement of sex chromosomes: XX/XY in mammalian females and males, and ZW/ZZ in avian females and males, respectively. There is evidence that genes on the sex chromosomes might influence sexual differentiation of the zebra finch song system [25,30,47-50]. The potential for sex chromosome genes to display sex differences in expression levels is especially high in the zebra finch because sex chromosome dosage compensation is largely ineffective in birds at the mRNA level [51-53].

Here, we examine 17β-hydroxysteroid dehydrogenase type 4 (HSD17B4), a steroidogenic enzyme that converts E_2 _to a less potent estrogen, estrone (E_1_). Inasmuch as HSD17B4 is present and active in particular brain regions, it could regulate the local concentrations of E_2_. This could impact the organization and function of the song circuit [12,13,24,50,54-56]. Previous experiments have shown evidence that HSD17B4 is localized to the Z chromosome and has a male-biased sex difference in brain expression levels across development [50,57]. HSD17B4 therefore represents a convergence of the steroidal and genetic theories of song system sexual differentiation. All of this also suggests, however, that E_2 _might be less available to the male brain, a prediction contrary to findings that E_2 _masculinizes the song system.

We therefore used several techniques to examine HSD17B4 DNA, mRNA, and enzyme activity in male and female zebra finch brains. We used posthatch day 5 (P5) and adult birds because at both ages, E_2 _meaningfully impacts the song system. P5 is within the period when steroid administration has the greatest masculinizing effect on song nuclei, other steroid-synthesizing genes are expressed in the brain at this early age, and it is just prior to the earliest emergence of identifiable song control nuclei [6,11,38,58,59]. Thus expression of HSD17B4 at this early age could alter the local steroid environment that contributes to the initial organization of song nuclei. In adulthood, measures in song nuclei show the potential for rapid and synaptic regulation of estradiol synthesis, which could have functional consequences for song production and perception [2-5]. Our results show that HSD17B4 is sexually dimorphic and could impact both developing and mature song nuclei, consistent with a previous report, and that dosage compensation of HSD17B4 gene may act at a post-transcriptional level [50]. It will require further study to understand how these dosage compensation mechanisms affect neural steroid production, and therefore organization and function of the sexually dimorphic song control circuit.

## Results

### Zebra finch HSD17B4 gene and cDNA sequences

**Figure 1:**
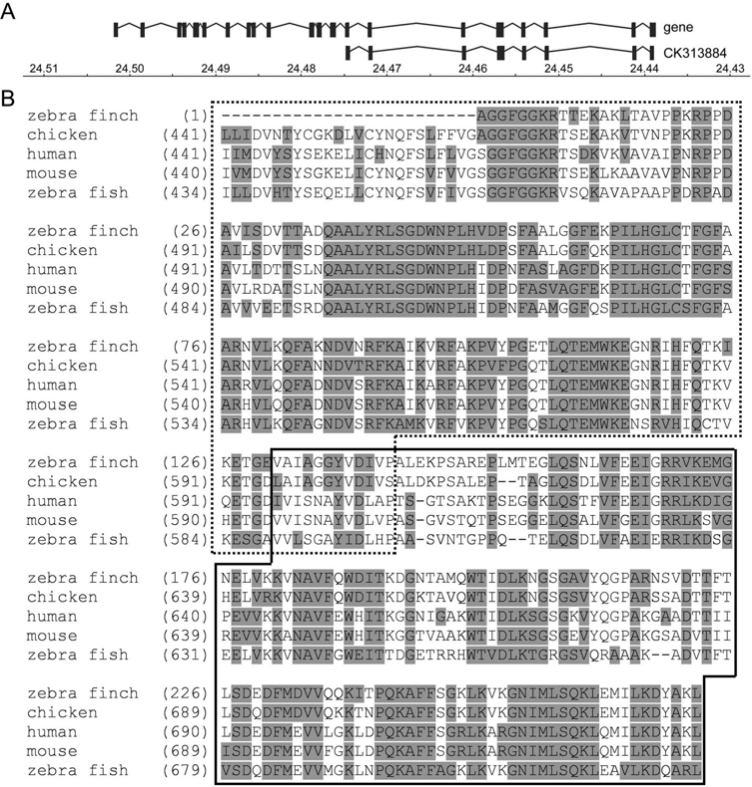
**Zebra finch HSD17B4 gene and cDNA clone identities**. (A) HSD17B4 gene model and EST structure based on the zebra finch genome assembly. The 5' end of the gene is to the left; a scale of the Z chromosome positions is on the bottom in 0.01 Mb increments. The gene model shows the coding exon structure. The zebra finch EST CK313884 is mapped below the entire gene model to show that the probes used in the study span approximately half of the gene at the 3' end. (B) Alignment of zebra finch EST CK313884 predicted protein sequence to that of chicken, human, mouse and zebra fish. Overall, there is high conservation of the amino acid sequence across species, including two functional domains: the hydratase-dehydrogenase epimerase (HDE, dashed box) domain and sterol carrier protein 2 (SCP2, solid box) domain, demonstrating the specificity of the probes used in molecular experiments.

We used two independently cloned cDNAs as probes for the molecular experiments. The clone used for Bacterial Artificial Clone (BAC) library screening, and Northern and Southern blot hybridizations was an Expressed Sequence Tag (EST; GenBank Accession No. CK313884) cloned by the Songbird Neurogenomics Initiative (SoNG) as part of its transcriptome project [60]. Based on alignment with the zebra finch HSD17B4 gene model (below), this cDNA includes sequence beginning at exon 14 through the end of the transcript and therefore spans part of the hydratase-dehydrogenase epimerase (HDE) domain, the entire sterol carrier protein 2 (SCP2) domain [61,62] and the 3'-UTR (Figure 1). The comparison of zebra finch, chicken, human, mouse and zebra fish predicted amino acid sequences showed that there is high conservation along HDE and SCP2 domains (range of 56%-90% identity across both domains for this subset of species comparisons; Figure 1).

Prior to the SoNG EST project, we had cloned a HSD17B4 cDNA that we used as the riboprobe template for our brain *in situ *hybridization experiments. The sequence of this clone spans almost exactly the same region of gene as the SoNG EST except that it begins 35 bp after the EST at the 5' end of the gene and extends 55 bp further than the EST at 3' end. The high level of cDNA sequence conservation across species, including essential functional domains, demonstrated that both cDNAs used in subsequent molecular experiments are almost certainly specific for HSD17B4.

To further confirm that the molecular tools we used were indeed HSD17B4, we examined the cDNA clone sequences in relation to the gene sequence in the zebra finch genome assembly. Using the Apollo Genome Annotation Curation Tool, we manually annotated the zebra finch DNA sequence from the genome assembly, the nucleotide sequences of the two clones we used for experiments, and other available transcript sequences to confirm the HSD17B4 gene model [63,64](Figure 1). In the zebra finch assembly, the gene was mapped to chromosome Z:24,439,644-24,502,076. As in the human gene, the zebra finch gene model contains 24 coding exons, although we were unable to predict the precise boundaries of the 5'-untranslated (UTR) region of the zebra finch gene using existing sequence data [65].

### Confirmation of Z chromosome localization

**Figure 2:**
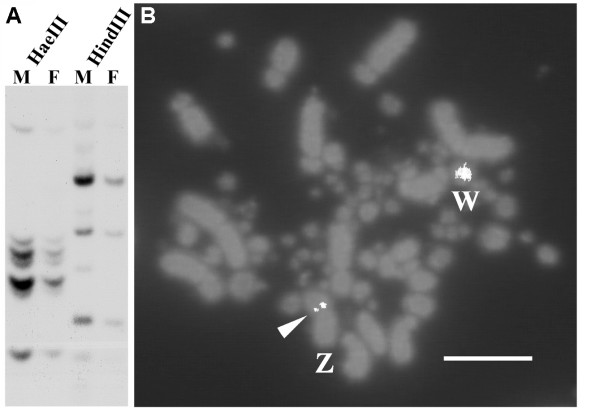
**Males have more Z chromosome-linked HSD17B4 DNA**. (A) Southern blot hybridization of HaeIII and HindIII digested genomic DNA from male (M) and female (F) of zebra finch probed with ^32^P-labeled zebra finch HSD17B4 EST CK313884. In both digest samples, the blot showed bands of stronger signal in males than females, suggesting Z chromosome linkage of HSD17B4. (B) Z chromosome localization of HSD17B4 sequence confirmed by FISH analysis. Individual fluorescently stained chromosomes are in the background, and two distinct, sharp hybridization signals over the Z chromosome show label on the two copies of the HSD17B4 gene (arrowhead). The Z-linked 094L04 BAC probe encoding HSD17B4 also cross-hybridized to the W chromosome, suggesting that it contains repetitive sequences such as ZBM that are present on both Z and W [67]. Scale bar = 10 μm.

We examined the intensity of hybridization on a Southern blot that contained male and female genomic DNA and to test if we could detect a sex difference in HSD17B4 gene abundance. To control for the possibility that gene products derived from one enzyme digestion could provide spurious results, we digested the genomic DNA with two enzymes, HaeII and HindIII, which results in two different banding patterns (Figure 2A). The male and female DNA showed virtually the same pattern of hybridized DNA fragments when digested with the same enzyme, with the exception of light-appearing bands that are visible in the male sample but not the female sample (Figure 2A). In cases when DNA products are visible in both male and female samples, the male bands appear to show darker hybridization signals than the corresponding female band. This suggests that the apparent sex difference in DNA digest products likely reflects the detection limit of the blot, not a biological difference in DNA content. The fact that hybridization showed stronger signals in the male DNA, almost twice the signal as from hybridization to the female DNA sample, suggests more copies of the gene in males than females (Figure 2A). The lack of a female-specific band in the Southern blot also suggests that the W chromosome does not have sequences homologous to CK313884.

To confirm the Z chromosome localization of HSD17B4 gene, we used the EST to first screen a zebra finch BAC library http://www.genome.arizona.edu to isolate a larger genomic clone to be used as the Fluorescent *In situ *Hybridization (FISH) probe [52]. The selected BAC clone, 094L04, encompassed HSD17B4 and mapped to the short arm of zebra finch Z chromosome by FISH (Figure 2B). The HSD17B4 BAC clone also cross-hybridized to the W chromosome, suggesting that the BAC clone contained the zebra finch ZBM (W chromosome repeat sequence)- related sequence [52,66,67].

### Northern blot hybridization shows sex difference in mRNA levels

**Figure 3:**
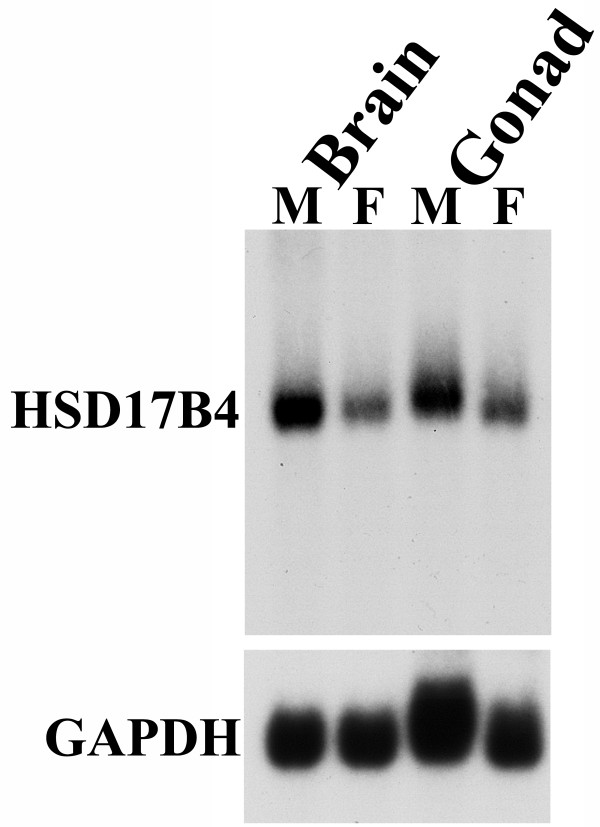
**Northern blot hybridization shows sex difference in HSD17B4 mRNA levels**. A Northern blot of brain and gonad total RNA from male (M) and female (F) zebra finches was hybridized with a ^32^P-labeled CK313884 probe. The blot shows that the band from the male brain sample is 1.7 times the intensity of that from the female brain sample, suggesting male higher expression of HSD17B4 mRNA. The control GAPDH hybridization indicates that the amount of male and female RNA loaded was roughly equivalent in the brain. The testicular and ovarian samples also show differential expression levels of HSD17B4, but GAPDH is likely expressed at higher levels in the testes than the ovaries, and therefore was not used as a loading control in these tissues [68].

Northern blot hybridization for male and female whole brain or gonads showed higher expression of HSD17B4 gene in males relative to females (Figure 3). In both the brain and gonads, the male:female ratio of the density of HSD17B4 hybridization was 1.7. In the brain samples, we could confirm that this sex bias was not due to overall differences in RNA levels loaded onto the blot; the male:female ratio of GAPDH control hybridization was 1.1. A similar control measure could not be obtained for the gonads because GAPDH expression levels can differ across tissues and the results here are consistent with previous reports that the zebra finch testes express higher levels of GAPDH than ovaries [68].

### Sex differences and song system localization of HSD17B4 mRNA

**Figure 4:**
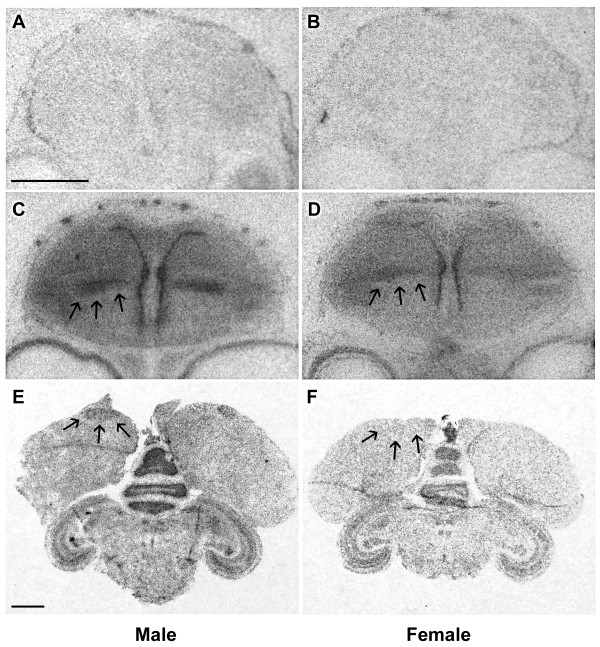
***In situ *hybridization for HSD17B4 in P5 and adult zebra finch song nuclei**. (A, B) Hybridization of P5 male (A) and P5 female (B) brain sections with sense-configured HSD17B4 riboprobe. Absence of detectable labeling demonstrates specificity of hybridization. (C, D) Comparison of HSD17B4 hybridization in P5 male (C) and female (D) brains with antisense-configured riboprobe shows label in the nidopallium where song nucleus LMAN (arrows) is located in adult males. In this region, HSD17B4 shows a significant (p = 0.021) sex difference in expression level. (E) HSD17B4 hybridization was also detected in the song nucleus HVC in adult males (arrows). (F) HVC was not visible in adult females (arrows denote area of HVC but no detectable hybridization), but the telencephalic levels of HSD17B4 expression were significantly (p = 0.016) higher in adult males than females. Scale bar in A = 1 mm (applies to A-D), scale bar in E = 1 mm (applies to E, F).

*In situ *hybridization confirmed the presence of HSD17B4 mRNA in the brains of both males and females, at P5 and in adulthood. Brain sections hybridized with the antisense probe showed widespread labeling of cells that express HSD17B4 (Figure 4). As expected, sections hybridized with the negative control sense-configured probes showed no label (Figure 4).

At P5, we detected HSD17B4 hybridization throughout the telencephalon and in the area that resembled the lateral magnocellular nucleus of the nidopallium (LMAN) in male and female brains. This was the only brain area that resembled a song nucleus that was visible at this young age, based on its neuroanatomical position and boundaries (Figure 4). There was no significant sex difference in hybridization density measurements of the entire telencephalon (p = 0.072). Within the presumptive LMAN region of P5 birds, however, t-tests showed there was a significant sex difference (male greater than female) in HSD17B4 hybridization intensity (p = 0.021).

We also detected HSD17B4 mRNA in the adult male HVC (used as a proper name) nucleus of the song circuit, though we were unable to perform a quantitative sex comparison because HVC was not reliably identified in comparable sections in females (Figure 4). Hybridization label in other song areas present in both males and females (LMAN and the robust nucleus of the arcopallium, RA) were not greater than the surrounding telencephalon. Therefore, we analyzed HSD17B4 hybridization intensities on the entire left and right telencephalic lobes in sections that spanned the entire rostral-caudal extent. This analysis revealed significantly higher hybridization levels in the telencephalon of males than of females (p = 0.016).

### HSD17B4 activity does not show a sex difference

**Figure 5:**
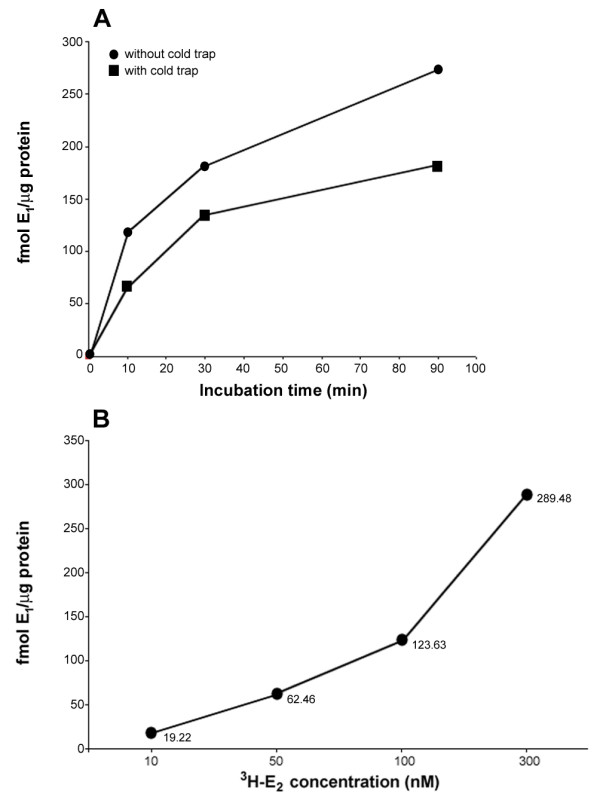
**HSD17B4 biochemical assay optimization**. (A) Results from timecourse experiment for biochemical meaures of HSD17B4 activity. Graph shows fmol E_1_/μg protein produced after brains were incubated for 10 minutes, 30 minutes or 90 minutes with and without cold trap. (B) Results from dose response curve for biochemical meaures of HSD17B4 activity. Graph shows fmol E_1_/μg protein produced after incubation of brain with 10 nM, 50 nM, 100 nM, 300 nM of ^3^H-E_2 _substrate for 5 minutes. Each point represents the average of duplicate measurements for each substrate concentration. 50 nM ^3^H-E_2 _substrate is centered in the linear rise of enzyme activity.

Several initial experiments were performed to first optimize the conditions for the final biochemical assessments of HSD17B4 activity in the brain. A timecourse experiment showed that a 5 minute incubation was optimal for accurately measuring HSD17B4 activity (Figure 5A). Presence of an E_1 _cold trap decreased the detectable conversion of E_2 _to E_1 _approximately two-fold (Figure 5B). Therefore, cold trap was not used in subsequent experiments. Lastly, a concentration gradient determined that 50 nM ^3^H-E_2 _was the optimum substrate concentration for measuring HSD17B4 activity after 5 minutes of incubation (Figure 5B).

**Figure 6:**
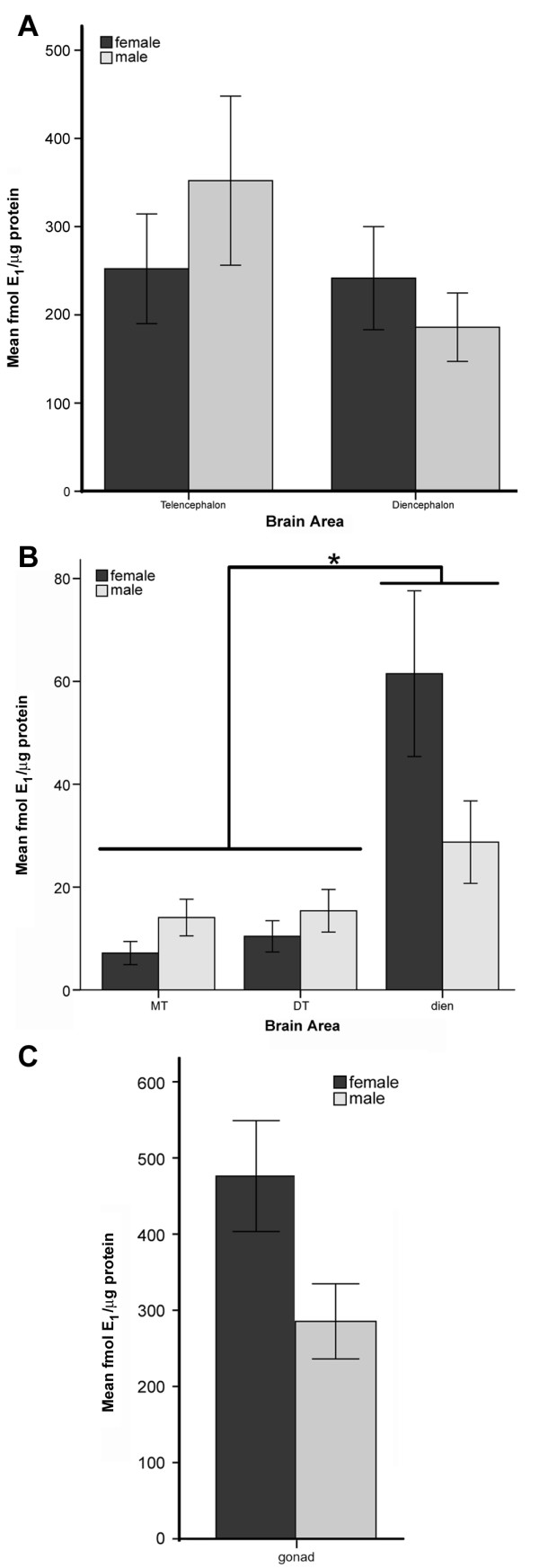
**Results of biochemical assays for HSD17B4 activity in male and female brain regions from two experiments**. (A) Comparison of HSD17B4 activity in whole telencephalon and diencephalon shows no significant differences between sex, brain region, or the interaction of sex by brain region. There is a subtle trend in the male towards higher activity in the telencephalon compared to male diencephalon and both female regions. (B) Comparison of HSD17B4 activity in two sub-regions of the telencephalon and the diencephalon showed no overall sex differences but a significant sex by brain area interaction (p = 0.017). (C) HSD17B4 activity levels measured in testes and ovaries are significantly higher than those in the brain (p < 0.001) but do not show a significant sex difference (p = 0.062). Error bars are ± s.e.m.

Using the optimized conditions, we first analyzed HSD17B4 activity in the telencephalon and diencephalon of adult male and female birds. Two-way ANOVA for sex, region and the sex by region interaction showed that there was no significant overall effect of sex (p = 0.745), brain region (p = 0.206), or the interaction (p = 0.263) on HSD17B4 activity levels (Figure 6A).

In a separate set of birds, we performed another assay for HSD17B4 activity in two telencephalic regions that contained brain regions that *in situ *hybridization and Northern blot results suggested would have HSD17B4 activity (dorsomedial telencephalon, "DT" and caudodorsal telencephalon, "CT"; details of the dissections in Methods), diencephalon, and gonads (Figure 6B, C). In this experiment, we found that the gonads contained significantly more HSD17B4 activity than the brain (p < 0.001). When the three brain areas were then analyzed for effects of age, sex, and their interaction, we found again that sex had no significant main effect on HSD17B4 activity levels (p = 0.323). Individual t-tests for the effect of sex within each of the three brain areas also did not detect a sex difference in any individual region (DT: p = 0.362, CT: p = 0.151, diencephalon: p = 0.106). In this assay, we did detect a significant main effect of brain area (p = 0.001). Post-hoc analysis demonstrated that the effect of brain area was due to higher levels of HSD17B4 activity in the diencephalon compared to the two telencephalic regions. Further, there was a significant interaction between sex and brain area (p = 0.043). The interaction occurred because in the diencephalon, we measured higher (but not significantly higher) HSD17B4 activity levels in the female (61.5 ± 36.0 6 fmol E_1_/μg protein) compared to the male (28.7 ± 18.0 6 fmol E_1_/μg protein) but in the two telencephalic regions, HSD17B4 activity levels were higher in the male (CT: 14.1 ± 7.1, DT: 15.4 ± 9.3 fmol E_1_/μg protein) than in the female (CT: 7.2 ± 4.5, DT: 10.4 ± 6.8 fmol E_1_/μg protein; Figure 6B).

## Discussion

Estradiol is a potent masculinizing steroid for the developmental growth of the sexually dimorphic song circuit in the zebra finch. Estradiol also continues to impact the function of song regions in adult birds [69]. Unlike most sexually dimorphic systems, gonadal secretions do not control song circuit masculinization. Consequently, two non-exclusive theories have been proposed to explain the sex difference in the song circuit: the neural production of steroids and the differential expression of sex chromosome genes in the brain. Here, we examined HSD17B4, an E_2_-metabolizing enzyme mapped to the Z sex chromosome, as an example of how these two theories might converge.

We confirmed the Z chromosome localization of the HSD17B4 gene, and that the abundance of HSD17B4 DNA and brain mRNA is approximately two-times greater in males a than in females, consistent with previous reports [50,57]. Based on *in situ *hybridization signal distributions here and in a previous study, it may be that HSD17B4 has a specific role in the sexual differentiation and function of the song circuit. Here, we showed that in the first week of posthatch development when E_2 _is most effective for masculinizing the song nuclei, HSD17B4 expression is detectable in the P5 brain and expression levels are higher in an area that appears to be LMAN in males than in females. If this is indeed LMAN, it would be the earliest identification of this nucleus in the zebra finch. Further, the LMAN expression appears to be developmentally regulated; in P25 birds, HSD17B4 hybridization intensity in LMAN was also higher than in the adjacent telencephalic tissue but there was no significant sex difference within the song nucleus, and by adulthood, label was not noticeably greater in LMAN than in the surrounding brain [50]. In contrast, we were able to detect HSD17B4 mRNA in the HVC of adult males (but not females), where HSD17B4 mRNA has been shown to be significantly enriched compared to the surrounding telencephalon in P25 and adult males [50,70]. This song nucleus also contains synaptic aromatase that can be modulated by song production, thus both aromatase and HSD17B4 may together regulate the availability of E_2 _in the adult HVC [2,4,39].

The higher levels of HSD17B4 expression in the male brain compared to the female brain, in particular within song control nuclei, were counterintuitive given extensive evidence that E_2 _masculinizes these areas [24]. Nevertheless, the higher expression of HSD17B4 mRNA in males than females was consistent with previous findings that many Z chromosome genes are poorly dosage compensated in birds; the majority of genes on the Z chromosome show higher mRNA levels in males (ZZ) than females (ZW) [51,66]. The incomplete dosage compensation in birds differs strikingly from the effective dosage compensation patterns found in other species [48,66]. It is generally accepted, however, that dosage compensation is critical and ubiquitous among species with heteromorphic sex chromosomes like the avian Z and W [48,71-73]. Thus, it was possible that dosage compensation could occur via post-transcriptional mechanisms in the zebra finch.

We therefore assayed the activity levels of HSD17B4 in adult brains. Our results demonstrate that this enzyme is active in metabolizing E_2 _in the zebra finch. However, in two major brain regions and in two experiments, the conversion of E_2 _to E_1 _was not significantly different between adult males and females - the sex difference in HSD17B4 mRNA levels in the adult brain does not translate into a sex difference in activity levels. Therefore, while the neuroanatomical mapping of HSD17B4 mRNA suggested that this enzyme may influence the sex-specific organization and function of the song control circuit by reducing the availability of E_2 _in males, our biochemical results indicate that post-transcriptional mechanisms may regulate neural HSD17B4 activity to minimize the sex difference found in HSD17B4 expression levels.

Perhaps regulatory mechanisms that are known to modify enzyme activity, such as phosphorylation and availability of substrate or cofactors, could explain our results. These factors might override the tendency for greater enzyme activity based on the male bias in mRNA abundance. Enzymes in particular have been theorized to be relatively dosage insensitive because their function is often more strongly dependent on properties other than the copy number of the genes encoding them [74]. Steroidogenic enzyme regulation may have been especially relevant to the evolution of an E_2_-sensitive neural circuit such as the zebra finch song system. The precise mechanisms that control, and the functional significance of, this region-specific regulation of HSD17B4 activity remains to be discovered.

## Conclusions

HSD17B4 is a Z-linked enzyme that shows a sex difference in DNA dose and mRNA expression levels, but not in activity levels. The post-transcriptional regulation of HSD17B4 may signal that E_2 _metabolism is tightly controlled in the brains of both male and female zebra finches.

## Methods

All zebra finches were obtained from colonies housed in the UCLA Life Science Vivarium. The Chancellor's Animal Research Committee approved all protocols.

### HSD17B4 gene and cDNA clone annotation

Two zebra finch HSD17B4 cDNA clones were independently produced and used for probe templates in subsequent experiments. We cloned one cDNA from zebra finch brain using PCR and plasmid cloning techniques as previously described [6]. Primers used to amplify the cDNA were: S: 5'-TTTTCCACCCAAGAGACCCC-3' AS: 5'-TGGCATAGTCTTTCAGGATCATTTC-3'. This clone was used as the riboprobe template for *in situ *hybridization experiments. After we had obtained this clone, the Songbird Neurogenomics Initiative (SoNG) cloned a HSD17B4 expressed sequence tag as part of a brain transcriptome project (EST; GenBank Accession No. CK313884) [60]. We obtained this clone and used it for the probe template for BAC library screening, Southern blot and Northern blot hybridizations. We compared the sequence of the two cDNA clones to determine that they largely overlap and therefore provided comparable measures of HSD17B4 DNA and mRNA across the experiments.

At the time both clones were produced, their identities as HSD17B4 sequences were based on homology matches to genes in other species such as human and chicken. To do this, we aligned the predicted amino acid sequence of the EST to the predicted amino acid sequence of the human, chicken, zebra fish and mouse HSD17B4 genes to assess the extent that the zebra finch sequence shares sequence and functional domain motifs with known HSD17B4 genes from other species.

Recently, the zebra finch genome has been sequenced and assembled [64]. Therefore, to further confirm the clone identities, we compared their nucleotide sequences to a model of the zebra finch gene sequence we constructed from the genome assembly. To create this model, we used DNA sequence from the zebra finch genome assembly, the EST sequence from the ESTIMA database and the sequence of our cloned cDNA, the set of RefSeq GenBank entries from other species, and the predicted human HSD17B4 protein coding sequence. With this information, we manually curated the HSD17B4 predicted gene sequence and structure in the Apollo Genome Annotation Curation Tool [63]. We then aligned the zebra finch brain cDNA clone sequences to the genomic sequence to identify the gene region included in the clones; we show the structural alignment of the gene to the SoNG EST sequence here.

### Southern blot hybridization

Zebra finch genomic DNA for Southern blot analysis was isolated from heparinized blood. Briefly, the nuclei of blood cells were suspended in 10 mM Tris-HCl (pH 8.0), 100 mM EDTA (pH 8.0) and incubated in the presence of 0.5% sodium dodecyl sulfate (SDS), 100 μg/ml proteinase K at 50°C overnight. The mixture was extracted successively with phenol saturated with TE [10 mM Tris-HCl (pH 8.0), 1 mM EDTA] and chloroform. The supernatant was ethanol precipitated, washed with 70% ethanol and resolved in TE. The purified genomic DNA was stored at 4°C until use.

Male or female genomic DNA was digested HindIII or HaeIII, then electrophoresed in a 1% agarose gel, 1× TAE (4 μg/lane). The gel was denatured with 0.5 M NaOH-1.5 M NaCl for 30 minutes, rinsed in water and neutralized in 1 M Tris (pH 7.6)-1.5 M NaCl for 30 minutes. After another wash in 20× SSC for 20 minutes, the gel was transferred to MAGNA Nylon Transfer membrane (Osmonics Inc, Minnetonka, MN) in 20× SSC overnight. The membrane was washed in 2× SSC, dried and baked at 80°C, then UV cross linked. The DNA for probes was agarose gel purified from restriction enzyme digested plasmids using the Gene Clean kit (Q-BIOgene, Solon, OH). Hybridization with a ^32^P-labeled DNA probe was carried out in a hybridization buffer [0.5 mol/L Na-phosphate buffer (pH 7.2), 7% SDS, 1 mmol/L EDTA, 1% BSA (A3059, Sigma, St. Louis, MO)] at 65°C overnight. The membrane was washed in 2× SSC, 0.1% SDS at room temperature for 1 minute twice and 65°C for 30 minutes twice, and then subjected to autoradiography.

Hybridization was performed with an expressed sequence tag (EST) clone from the ESTIMA database (GenBank Accession No. CK313884) that was identified as HSD17B4. Before using CK313884 as a probe in any of these experiments, we first confirmed its identity by comparing it to known HSD17B4 sequences in other species and confirming known conserved functional domains (MOTIF, http://motif.genome.jp/.

### Fluorescent *in situ *hybridization (FISH)

The HSD17B4 zebra finch bacterial artificial chromosome (BAC) clone, 094L04, was isolated from a zebra finch BAC library made by the Arizona Genomics Institute http://www.genome.arizona.edu after hybridization with CK313884 probe. Isolation of BAC DNA, chromosome preparation and FISH to mitotic chromosomes prepared from zebra finch fibroblasts were carried out according to the methods of Itoh and Arnold [75].

### Northern blot hybridization

RNAs for male and female adult zebra finch brain or gonads were isolated using Trizol (Invitrogen, Carlsbad, CA) according to manufacturer's instructions, and stored at -80°C until use. RNA samples (8 μg/lane) were subjected to denaturing agarose gel electrophoresis and capillary transferred to a MAGNA Nylon Transfer membrane (Osmonics Inc) in 10× SSC. The membrane was hybridized with a ^32^P-labeled probe based on CK313884, in the same hybridization buffer used for Southern blot hybridization (above), at 68°C for 12 hours. After hybridization, the membrane was washed in 2× SSC, 0.1% SDS at room temperature for 5 minutes twice, 1× SSC, 0.1% SDS at 37°C for 15 minutes twice and 65°C for 15 minutes twice, and finally subjected to autoradiography. After hybridization for HSD17B4, the blots were stripped and re-hybridized for GAPDH as an RNA loading control.

The density of the bands for brain and gonad samples was quantified in Image J http://rsbweb.nih.gov/ij/. The density of the region of the blot surrounding the band was subtracted from the density of the hybridized band to normalize for any differences in background. This process was performed for bands hybridized with the HSD17B4 probe and the GAPDH probe. We report male:female density ratios except in the case of GADPH hybridization to the gonads, which was higher in testes than in ovaries, as in a previous report [68].

### *In situ *hybridization and analysis

P5 and adult brains were collected, flash frozen, and stored at -80°C until use. Delicate P5 brains were retained in the skull to prevent damage from dissection. Both male and female brains from each age (n = 4 per sex for P5, n = 6 per sex for adults) were sectioned to 20 μm for *in situ *hybridization. The sex of P5 birds was verified with visual inspection of the gonads. Prior to hybridization, slides were dried at room temperature for 1 hour, then processed as previously described in London et al., 2003 [6]. Male and female sections from each age were processed together to facilitate quantitative comparisons of male and female hybridization levels at P5 and adult. Slides were hybridized overnight at 60°C with 4 × 10^6 ^cpm of either antisense- or sense- configured ^33^P-UTP labeled riboprobes. The probe template was a 761 bp zebra finch HSD17B4 clone independently created from the EST described above, but that overlaps with nucleotides 36-796 of the CK313884 sequence.

After hybridization, slides were washed and dehydrated as previously described [6]. The high stringency wash was performed at 63°C for 30 minutes. Slides were dried at room temperature at least 2 hours, then exposed to Kodak MR film (Kodak, Rochester, NY). Film was exposed for ~17 hours for adult brains, ~4 days for P5 brains at room temperature, before developing. Slides were subsequently dipped in liquid photographic emulsion, developed 9-10 days later, dehydrated and coverslipped.

To quantify optical density of select brain regions, digital pictures of film exposures were first converted to grayscale in Adobe Photoshop, then the average density of each region was measured in Scion Image http://www.scioncorp.com/pages/scion_image_windows.htm. Background density was also measured and subtracted from the original density, giving the overall mean density. Quantification of telencephalic hybridization intensities was performed on a series of sections approximately 100 μm apart that spanned the rostral to caudal extent of the telencephalon; the entire lobe of the telencephalon was included from both left and right hemispheres in all sections that contained telencephalon. This data was then analyzed with t-tests (SSPS, SSPS Inc., Chicago, IL) to test for significant sex differences in hybridization intensities.

### Image production

*In situ *hybridization images from BioMax MR films were captured with a Zeiss Stemi 2000-C dissecting scope mounted with an AxioCam MRc digital camera with MRGrab 1.0 software (Carl Zeiss Inc., Thornwood, NY). Southern and Northern blot film images were captured by scanning into an EPSON Expression 800 flatbed scanner.

### HSD17B4 activity

To measure HSD17B4 activity in brain tissues, we used the core biochemical procedure previously described for other steroidogenic enzymes [19,22,30,39,40,76-78]. Briefly, tissue was homogenized in sucrose phosphate buffer (pH 9.0). Aliquots from the homogenates are added to 3 mM nicotinamide adenine dinucleiotide (NAD+) cofactor and tritiated E_2_(^3^H-E_2_) substrate, and incubated at 41°C. Enzymatic activity was stopped by flash freezing. Steroids were purified from proteins via repeated ether extractions. To separate individual steroids, samples were spotted on thin layer chromatography (TLC) plates after the addition of non-radiolabeled E_1 _and E_2 _to facilitate steroid visualization. TLC plates were resolved in a 3:1 ether:hexane mixture. Separated bands identified as E_1 _and E_2 _from standard samples were scraped from the TLC plates, eluted into methanol, and ^3^H-estrogens were directly quantified in a liquid scintillation counter. To normalize the quantity of steroid produced to the mass of protein in each sample, a Bradford protein assay was performed with the protein fraction of the homogenates. All sample tubes were run in triplicate, and "S" tubes that contained no tissue and an "R" tube that contained only ^3^H-E_2 _were included to assay background levels of metabolite and percent recovery of steroids, respectively. The total quantity of ^3^H-E_1 _synthesized in each reaction was calculated by subtracting background values from the total ^3^H-E_1 _counts per minute (cpm) for each sample, and dividing this value by the percent recovery of each assay. Values are reported as femtomoles of ^3^H-steroid per microgram of protein (fmol E_1_/μg protein).

Since the activity levels of HSD17B4 enzyme have not previously been studied in the zebra finch, we first performed several experiments to optimize conditions for its measurement. To determine the most accurate period of tissue incubation, we performed a timecourse experiment. We used two adult female whole brains, and after homogenization, samples were incubated with 100 nM ^3^H-E_2 _and incubated at 41°C for 10, 30, or 90 minutes. Results were plotted to determine a time of linear activity levels. To determine if the addition of an E_1 _"cold trap," radioinert E_1_, would enhance the detection of ^3^H-E_1 _by limiting the conversion of ^3^H-E_1 _to other steroid products, 1 μg E_1 _was added to half of the tubes in a preliminary experiment. Lastly, to determine the optimal ^3^H-E_2 _concentration to use for substrate, a female whole brain was incubated in the presence of 10, 50, 100, or 300 nM ^3^H-E_2 _and processed as above.

Utilizing the conditions determined by preliminary experiments (5 minute incubation with 100 nM ^3^H-E_2 _and no cold trap), we then performed two experiments to measure the HSD17B4 activity in dissected brain areas. First, we assayed HSD17B4 levels in the telencephalon and diencephalon of adult males and females (n = 5 per sex). Second, we further dissected the telencephalon into two parts: the dorsomedial telencephalon ("DT," tissue between the midline and 2 mm lateral to midline starting 5 mm from the rostral tip of the brain and terminating 5 mm from the caudal end of the telencephalon, a region that contains the hippocampus and lateral ventricles), and the caudodorsal telencephalon ("CT," a 1.5 mm deep section containing the caudal half of the dorsal telencephalon that contains HVC). We also assayed the diencephalon, ovaries and testes from each bird (n = 5 per sex, except CT n = 4 per sex due to technical issues). One- and two-way ANOVA and Tukey post-hoc tests (SPSS) were used to test for effects of sex and brain region on HSD17B4 activity.

## Authors' contributions

SEL designed experiments, interpreted data, wrote the manuscript and created figures; YI designed, conducted, and interpreted experiments, wrote the manuscript and created figures; VAL, PW, PSE, and RKO conducted experiments; APA, and BAS designed experiments, interpreted data, and wrote the manuscript. All authors read and approved the manuscript.

## References

[B1] Nottebohm F Arnold AP Sexual dimorphism in vocal control areas of the songbird brain Science 1976 194 211 213 10.1126/science.959852 959852

[B2] Peterson RS Yarram L Schlinger BA Saldanha CJ Aromatase is pre-synaptic and sexually dimorphic in the adult zebra finch brain Proc Royal Society B: Biol Sci 2005 272 2089 2096 10.1098/rspb.2005.3181 PMC1559905 16191621

[B3] Remage-Healey L Maidment NT Schlinger BA Forebrain steroid levels fluctuate rapidly during social interactions Nat Neurosci 2008 11 1327 1334 10.1038/nn.2200 18820691 PMC2577388

[B4] Remage-Healey L Oyama RK Schlinger BA Elevated aromatase activity in forebrain synaptic terminals during song J Neuroendocrinol 2009 21 191 199 10.1111/j.1365-2826.2009.01820.x 19207827 PMC2680705

[B5] Rohmann KN Schlinger BA Saldanha CJ Subcellular compartmentalization of aromatase is sexually dimorphic in the adult zebra finch brain Dev Neurobiol 2007 67 1 9 10.1002/dneu.20303 17443767

[B6] London SE Boulter J Schlinger BA Cloning of the zebra finch androgen synthetic enzyme CYP17: a study of its neural expression throughout posthatch development J Comp Neurol 2003 467 496 508 10.1002/cne.10936 14624484

[B7] London SE Monks DA Wade J Schlinger BA Widespread capacity for steroid synthesis in the avian brain and song system Endocrinol 2006 147 5975 5987 10.1210/en.2006-0154 PMC2903432 16935847

[B8] Holloway CC Clayton DF Estrogen synthesis in the male brain triggers development of the avian song control pathway in vitro Nat Neurosci 2001 4 170 175 10.1038/84001 11175878

[B9] Grisham W Mathews GA Arnold AP Local intracerebral implants of estrogen masculinize some aspects of the zebra finch song system J Neurobiol 1994 25 185 196 10.1002/neu.480250209 8021647

[B10] Jacobs EC Arnold AP Campagnoni AT Developmental regulation of the distribution of aromatase- and estrogen-receptor-mRNA-expressing cells in the zebra finch brain Dev Neurosci 2000 21 453 472 10.1159/000017413 10640864

[B11] London SE Schlinger BA Steroidogenic enzymes along the ventricular proliferative zone in the developing songbird brain J Comp Neurol 2007 502 507 521 10.1002/cne.21335 17394140

[B12] London SE Remage-Healey L Schlinger BA Neurosteroid production in the songbird brain: A re-evaluation of core principles Front Neuroendocrinol 2009 30 302 314 10.1016/j.yfrne.2009.05.001 19442685 PMC2724309

[B13] Remage-Healey L London SE Schlinger BA Birdsong and the neural production of steroids J Chem Neuroanatomy 2010 39 72 81 10.1016/j.jchemneu.2009.06.009 PMC2821977 19589382

[B14] Saldanha CJ Tuerk MJ Kim YH Fernandes AO Arnold AP Schlinger BA Distribution and regulation of telencephalic aromatase expression in the zebra finch revealed with a specific antibody J Comp Neurol 2000 423 619 630 10.1002/1096-9861(20000807)423:4<619::AID-CNE7>3.0.CO;2-U 10880992

[B15] Schlinger BA Arnold AP Brain is the major site of estrogen synthesis in a male songbird Proc Natl Acad Sci USA 1991 88 4191 4194 10.1073/pnas.88.10.4191 2034664 PMC51624

[B16] Schlinger BA Arnold AP Circulating estrogens in a male songbird originate in the brain Proc Natl Acad Sci USA 1992 89 7650 7653 10.1073/pnas.89.16.7650 1502177 PMC49768

[B17] Schlinger BA Soma KK London SE Neurosteroids and brain sexual differentiation Trends Neurosci 2001 24 429 431 10.1016/S0166-2236(00)01855-5 11476868

[B18] Shen P Campagnoni CW Kampf K Schlinger BA Arnold AP Campagnoni AT Isolation and characterization of a zebra finch aromatase cDNA: In situ hybridization reveals high aromatase expression in brain Mol Brain Res 1994 24 227 237 10.1016/0169-328X(94)90136-8 7968362

[B19] Tam H Schlinger BA Activities of 3beta-HSD and aromatase in slices of developing and adult zebra finch brain Gen Comp Endocrinol 2007 150 26 33 10.1016/j.ygcen.2006.07.001 16919626 PMC2724308

[B20] Gong A Freking FW Wingfield J Schlinger BA Arnold AP Effects of embryonic treatment with fadrozole on phenotype of gonads, syrinx, and neural song system in zebra finches Gen Comp Endocrinol 1999 115 346 353 10.1006/gcen.1999.7318 10480985

[B21] Wade J Springer ML Wingfield JC Arnold AP Neither testicular androgens nor embryonic aromatase activity alters morphology of the neural song system in zebra finches Biol Reproduction 1996 55 1126 1132 10.1095/biolreprod55.5.1126 8902226

[B22] Wade J Arnold AP Functional testicular tissue does not masculinize development of the zebra finch song system Proc Natl Acad Sci USA 1996 93 5264 5268 10.1073/pnas.93.11.5264 8643564 PMC39233

[B23] Wade J Swender DA McElhinny TL Sexual differentiation of the zebra finch song system parallels genetic, not gonadal, sex Horm Behav 1999 36 141 152 10.1006/hbeh.1999.1537 10506538

[B24] Wade J Arnold AP Sexual differentiation of the zebra finch song system Ann N Y Aca Sci 2004 1016 540 559 10.1196/annals.1298.015 15313794

[B25] Agate RJ Grisham W Wade J Mann S Wingfield J Schanen C Palotie A Arnold AP Neural, not gonadal, origin of brain sex differences in a gynandromorphic finch Proc Natl Acad Sci USA 2003 100 4873 4878 10.1073/pnas.0636925100 12672961 PMC153648

[B26] Grisham W Tam A Greco CM Schlinger BA Arnold AP A putative 5 alpha-reductase inhibitor demasculinizes portions of the zebra finch song system Brain Research 1997 750 122 128 10.1016/S0006-8993(96)01336-4 9098536

[B27] Grisham W Lee J McCormick ME Yang-Stayner K Arnold AP Antiandrogen blocks estrogen-induced masculinization of the song system in female zebra finches J Neurobiol 2002 51 1 8 10.1002/neu.10028 11920723

[B28] Mathews GA Arnold AP Antiestrogens fail to prevent the masculine ontogeny of the zebra finch song system Gen Comp Endocrinol 1990 80 48 58 10.1016/0016-6480(90)90147-E 2272479

[B29] Arnold AP Sex chromosomes and brain gender Nat Rev Neurosci 2004 5 701 708 10.1038/nrn1494 15322528

[B30] Chen X Agate RJ Itoh Y Arnold AP Sexually dimorphic expression of trkB, a Z-linked gene, in early posthatch zebra finch brain Proc Natl Acad Sci USA 2005 102 7730 7735 10.1073/pnas.0408350102 15894627 PMC1140405

[B31] Cam V Schlinger BA Activities of aromatase and 3beta-hydroxysteroid dehydrogenase/delta4-delta5 isomerase in whole organ cultures of tissues from developing zebra finches Horm Behav 1998 33 31 39 10.1006/hbeh.1998.1434 9571011

[B32] Jacobs EC Arnold AP Campagnoni AT Zebra finch estrogen receptor cDNA: cloning and mRNA expression J Steroid Biochem and Mol Biol 1996 59 135 145 10.1016/S0960-0760(96)00096-9 9010328

[B33] Kim YH Perlman WR Arnold AP Expression of androgen receptor mRNA in zebra finch song system: developmental regulation by estrogen J Comp Neurol 2004 469 535 547 10.1002/cne.11033 14755534

[B34] Perlman WR Arnold AP Expression of estrogen receptor and aromatase mRNAs in embryonic and posthatch zebra finch brain J Neurobiol 2003 55 204 219 10.1002/neu.10190 12672018

[B35] Perlman WR Ramachandran B Arnold AP Expression of androgen receptor mRNA in the late embryonic and early posthatch zebra finch brain J Comp Neurol 2003 455 513 530 10.1002/cne.10510 12508324

[B36] Schlinger BA Amur-Umarjee S Campagnoni AT Arnold AP 5 beta-reductase and other androgen-metabolizing enzymes in primary cultures of developing zebra finch telencephalon J Neuroendocrinol 1995 7 187 192 10.1111/j.1365-2826.1995.tb00746.x 7606244

[B37] Wade J Schlinger BA Arnold AP Aromatase and 5 beta-reductase activity in cultures of developing zebra finch brain: an investigation of sex and regional differences J Neurobiol 1995 27 240 251 10.1002/neu.480270210 7658203

[B38] Adkins-Regan E Mansukhani V Seiwert C Thompson R Sexual differentiation of brain and behavior in the zebra finch: Critical periods for effects of early estrogen treatment J Neurobiol 1994 25 865 877 10.1002/neu.480250710 8089662

[B39] Rohmann KN Schlinger BA Saldanha CJ Subcellular compartmentalization of aromatase is sexually dimorphic in the adult zebra finch brain J Neurobiol 2006 67 1 9 10.1002/neu.20303 17443767

[B40] Simpson HB Vicario DS Early estrogen treatment alone causes female zebra finches to produce learned, male-like vocalizations J Neurobiol 1991 22 755 776 10.1002/neu.480220710 1765782

[B41] Simpson HB Vicario DS Early estrogen treatment of female zebra finches masculinizes the brain pathway for learned vocalizations J Neurobiol 1991 22 777 793 10.1002/neu.480220711 1765783

[B42] Wade J Arnold AP Post-hatching inhibition of aromatase activity does not alter sexual differentiation of the zebra finch song system Brain Res 1994 639 347 350 10.1016/0006-8993(94)91752-3 8205488

[B43] Grisham W Park SH Hsia JK Kim C Leung MC Kim L Arnold AP Effects of long-term flutamide treatment during development in zebra finches Neurosci Letters 2007 418 92 96 10.1016/j.neulet.2007.03.002 PMC2169516 17398002

[B44] Mathews GA Brenowitz EA Arnold AP Paradoxical hypermasculinization of the zebra finch song system by an antiestrogen Horm Behav 1988 22 540 551 10.1016/0018-506X(88)90057-8 3235068

[B45] Schlinger BA Arnold AP Androgen effects on the development of the zebra finch song system Brain Res 1991 561 99 105 10.1016/0006-8993(91)90754-J 1797353

[B46] Wade J Springer ML Wingfield JC Arnold AP Neither testicular androgens nor embryonic aromatase activity alters morphology of the neural song system in zebra finches Biol Reprod 1996 55 1126 1132 10.1095/biolreprod55.5.1126 8902226

[B47] Agate RJ Choe M Arnold AP Sex differences in structure and expression of the sex chromosome genes CHD1Z and CHD1W in zebra finches Mol Biol Evol 2004 21 384 396 10.1093/molbev/msh027 14660691

[B48] Arnold AP Itoh Y Melamed E A Birds-Eye View of Sex Chromosome Dosage Compensation Annu Rev Genomics Hum Genet 2008 9 109 127 10.1146/annurev.genom.9.081307.164220 18489256

[B49] Wade J Tang YP Peabody C Tempelman RJ Enhanced gene expression in the forebrain of hatchling and juvenile male zebra finches J Neurobiol 2005 64 224 238 10.1002/neu.20141 15849735

[B50] Tomaszycki ML Peabody C Replogle K Clayton DF Tempelman RJ Wade J Sexual differentiation of the zebra finch song system: Potential roles for sex chromosome genes BMC Neurosci 2009 10 24 10.1186/1471-2202-10-24 19309515 PMC2664819

[B51] Ellegren H Hultin-Rosenberg L Brunstrom B Dencker L Kultima K Scholz B Faced with inequality: chicken do not have a general dosage compensation of sex-linked genes BMC Biol 2007 5 40 10.1186/1741-7007-5-40 17883843 PMC2099419

[B52] Itoh Y Kampf K Arnold AP Comparison of the chicken and zebra finch Z chromosomes shows evolutionary rearrangements Chromosome Res 2006 14 805 815 10.1007/s10577-006-1082-1 17139532

[B53] Melamed E Arnold AP Regional differences in dosage compensation on the chicken Z chromosome Genome Biol 2007 8 R202 10.1186/gb-2007-8-9-r202 17900367 PMC2375040

[B54] Forlano PM Schlinger BA Bass AH Brain aromatase: new lessons from non-mammalian model systems Front Neuroendocrinol 2006 27 247 274 10.1016/j.yfrne.2006.05.002 16828853

[B55] Hojo Y Murakami G Mukai H Higo S Hatanaka Y Ogiue-Ikeda M Ishii H Kimoto T Kawato S Estrogen synthesis in the brain--role in synaptic plasticity and memory Mol Cell Endocrinol 2008 290 31 43 10.1016/j.mce.2008.04.017 18541362

[B56] Woolley CS Estrogen-mediated structural and functional synaptic plasticity in the female rat hippocampus Horm Behav 1998 34 140 148 10.1006/hbeh.1998.1466 9799624

[B57] Itoh Y Kampf K Arnold AP Comparison of the chicken and zebra finch Z chromosomes shows evolutionary rearrangements Chrom Res 2006 14 805 815 10.1007/s10577-006-1082-1 17139532

[B58] Gahr M Metzdorf R The sexually dimorphic expression of androgen receptors in the song nucleus hyperstriatalis ventrale pars caudale of the zebra finch develops independently of gonadal steroids J Neurosci 1999 19 2628 2636 10087076 10.1523/JNEUROSCI.19-07-02628.1999 PMC6786054

[B59] Kim YH Perlman WR Arnold AP Expression of Androgen Receptor mRNA in Zebra Finch Song System: Developmental Regulation by Estrogen J Comp Neurol 2004 469 535 547 10.1002/cne.11033 14755534

[B60] Replogle K Arnold AP Ball GF Band M Bensch S Brenowitz Dong S Drnevich J Ferris M George JM Gong G Hasselquist D Hernandez AG Kim R Lewin HA Liu L Lovell PV Mello CV Naurin S Rodriguez-Zas S Thimmapuram J Wade J Clayton DF The Songbird Neurogenomics (SoNG) Initiative: Community-based tools and strategies for study of brain gene function and evolution BMC Genomics 2008 9 134 10.1186/1471-2164-9-131 18366674 PMC2329646

[B61] Adamski J Leenders F Carstensen JF Kaufmann M Markus MM Husen B Tesdorpf JG Seedorf U de Launoit Y Jakob F Steroids, fatty acyl-CoA, and sterols are substrates of 80-kDa multifunctional protein Steroids 1997 62 159 163 10.1016/S0039-128X(96)00175-4 9029731

[B62] Normand T Husen B Leenders F Pelczar H Baert JL Begue A Flourens AC Adamski J de Launoit Y Molecular characterization of mouse 17 beta-hydroxysteroid dehydrogenase IV J Steroid Biochem Mol Biol 1995 55 541 548 10.1016/0960-0760(95)00204-9 8547180

[B63] Lewis SE Searle SM Harris N Gibson M Lyer V Richter J Wiel C Bayraktaroglir L Birney E Crosby MA Kaminker JS Matthews BB Prochnik SE Smithy CD Tupy JL Rubin GM Misra S Mungall CJ Clamp ME Apollo: a sequence annotation editor Genome Biol 2002 3 RESEARCH0082 10.1186/gb-2002-3-12-research0082 12537571 PMC151184

[B64] Warren WC Clayton DF Ellegren H Arnold AP Hillier LW Kunstner A Searle S White S Vilella AJ Heger A Kong L Ponting CP Jarvis E Mello CV Minx P Yang S-P Lovell P Velho TAF Ferris M Balakrishnan CN Sinha S Blatti C London SE Li Y Lin Y-C George J Sweedler J Southey B Gunaratne P Watson M Nam K Backstrom N Smeds L Nabholz B Itoh Y Howard J Pffenning A Whitney O Völker M Skinner BM Griffin DK Ye L Flicek P Quesada V Velasco G Lopez-Otin C Puente XS Oleander T Lancet D Villela A Smit AFA Hubley R Konkel M Walker JA Batzer MA Gu W Pollock DD Chen L Cheng G Eichler E Stapley J Slate J Ekblom R Burt D Scharff C Adam I Richard H Sultan M Soldatov A Graves T Fulton L Nelson J Chinwalla A Hou S Mardis ER Wilson RK The genome of a songbird Nature in press 10.1038/nature08819 PMC3187626 20360741

[B65] Leenders F Dolez V Begue A Moller G Gloeckner JC de Launoit Adamski J Structure of the gene for the human 17beta-hydroxysteroid dehydrogenase type IV Mamm Genome 1998 9 1036 1041 10.1007/s003359900921 9880674

[B66] Itoh Y Melamed E Yang X Kampf K Wang S Yehya N Van Nas A Replogle K Band MR Clayton DF Schadt EE Lusis AJ Arnold AP Dosage compensation is less effective in birds than in mammals J Biol 2007 6 2 10.1186/jbiol53 17352797 PMC2373894

[B67] Itoh Y Kampf K Arnold AP Molecular cloning of zebra finch W chromosome repetitive sequences: Evolution of the avian W chromosome Chromosoma 2008 117 111 121 10.1007/s00412-007-0130-8 17972090

[B68] Itoh Y Kampf K Arnold AP Disruption of FEM1C-W gene in zebra finch: Evolutionary insights on avian ZW genes Chromosoma 2009 118 323 334 10.1007/s00412-008-0199-8 19139913

[B69] Remage-Healey L Coleman MJ Oyama RK Schlinger BA Brain estrogens rapidly strengthen auditory encoding and guide song preference in a songbird Proc Natl Acad Sci USA 107 8 3852 7 10.1073/pnas.0906572107 20133597 PMC2840459

[B70] Lovell PV Clayton DF Replogle KL Mello CV Birdsong "transcriptomics": Neurochemical specializations of the oscine song system PLoS ONE 2008 3 e3440 10.1371/journal.pone.0003440 18941504 PMC2563692

[B71] Gilfillan GD Straub T de Wit E Greil F Lamm R van Steensel B Chromosome-wide gene-specific targeting of the Drosophila dosage compensation complex Genes Dev 2006 20 858 870 10.1101/gad.1399406 16547172 PMC1475731

[B72] Gupta V Parisi M Sturgill D Nuttall R Doctolero M Dudko OK Malley JD Eastman PS Oliver B Global analysis of X-chromosome dosage compensation J Biol 2006 5 3 10.1186/jbiol30 16507155 PMC1414069

[B73] Nguyen DK Disteche CM Dosage compensation of the active X chromosome in mammals Nat Genetics 2006 38 47 53 10.1038/ng1705 16341221

[B74] Kacser H Burns JA The molecular basis of dominance Genetics 1981 97 639 666 7297851 10.1093/genetics/97.3-4.639 PMC1214416

[B75] Itoh Y Arnold AP Chromosomal polymorphism and comparative painting analysis in the zebra finch Chrom Res 2005 13 47 56 10.1007/s10577-005-6602-x 15791411

[B76] Cam V Schlinger BA Activities of aromatase and 3β-hydroxysteroid dehydrogenase/Δ4-Δ5 isomerase in whole organ cultures of tissues from developing zebra finches Horm Behav 1998 33 31 39 10.1006/hbeh.1998.1434 9571011

[B77] Freking F Nazairians T Schlinger BA The expression of the sex steroid-synthesizing enzymes CYP11A1, 3beta-HSD, CYP17, and CYP19 in gonads and adrenals of adult and developing zebra finches Gen Comp Endocrinol 2000 119 140 151 10.1006/gcen.2000.7503 10936034

[B78] Schlinger BA Lane NI Grisham W Thompson L Androgen synthesis in a songbird: a study of cyp17 (17alpha-hydroxylase/C17,20-lyase) activity in the zebra finch Gen Comp Endocrinol 1999 113 46 58 10.1006/gcen.1998.7179 9882543

